# INI-1-Deficient Sinonasal Carcinoma: Case Report with Emphasis on Differential Diagnosis

**DOI:** 10.1155/2022/5629984

**Published:** 2022-03-30

**Authors:** Anwaar M. Alsayed, Eman A. Aljufairi, Amjad O. Alshammari, Khalid A. Alsindi, Omar A. Sabra

**Affiliations:** ^1^Department of Histopathology, King Hamad University Hospital, Bahrain; ^2^Department of Ear, Nose and Throat, King Hamad University Hospital, Bahrain

## Abstract

SMARCB1-deficient sinonasal carcinoma is a newly described entity, with less than 100 reported cases. It is characterized by basaloid or rhabdoid morphology and is diagnosed by complete loss of nuclear SMARCB1 (INI-1). The morphologic appearance, specific immunophenotypic markers, and unique molecular make-up distinguish this entity from other various malignant neoplasms. We present a case of a 55-year-old male that presented with a large progressing palatine mass. Magnetic resonance imaging showed a heterogeneous mass involving the left maxillary space. The initial biopsy was diagnosed as undifferentiated carcinoma. Resection was performed, and immunohistochemical studies revealed a complete loss of INI-1, refining the diagnosis to SMARCB1-deficient sinonasal carcinoma. Diagnosis of SMARCB1-deficient sinonasal carcinoma should be considered in all undifferentiated sinonasal carcinomas. Immunohistochemistry or molecular studies are mandatory to confirm the diagnosis and exclude other morphologically similar entities.

## 1. Introduction

SMARCB1-deficient sinonasal carcinoma is a rare, aggressive, poorly differentiated carcinoma of the sinonasal tract. It is defined by the complete loss of nuclear SMARCB1 (INI-1) expression by immunohistochemistry or cytogenetic abnormalities within the 22q11.2 chromosome [1–5].

SMARCB1-deficient sinonasal carcinoma is a relatively new entity with under 100 cases being reported to date [6]. In this paper, we present a case of SMARCB1-deficient sinonasal carcinoma of the maxillary sinus in a 55-year-old male, which was initially diagnosed as sinonasal undifferentiated carcinoma.

## 2. Case Presentation

A 55-year-old man presented with a 2-month history of a painless, progressively enlarging mass on the left palate along with a left-sided nasal blockage, facial pain, and intermittent epistaxis. He smoked one pack of cigarettes a day for the past 18 years and declared no alcohol consumption.

Clinical examination revealed a left-sided facial swelling and a left cervical lymphadenopathy. This swelling was seen as a frail vascular mass on endoscopic examination, almost filling the left nasal cavity with further extension inferiorly to the left palate.

**Figure 1:**
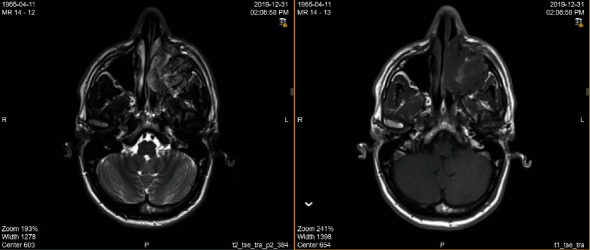
Orbital MRI images show a large mass occupying and destructing the left maxillary sinus with extension into the left nasal cavity and the adjacent retromaxillary space.

Magnetic resonance imaging (MRI) scan disclosed a heterogeneous appearance of this paranasal sinus mass, with further involvement of the left maxillary space, nasal cavity, destructive extension of the hard palate, retromaxillary space, and premaxillary soft tissue. No intracranial or intraorbital extension was found. Additionally, left upper deep cervical, submental, and submandibular pathological necrotic lymph nodes were spotted as well (Figure 1).

Biopsies from the mass and cervical lymph node were taken and reported as undifferentiated carcinoma. The patient underwent total maxillectomy with reconstruction and bilateral neck dissection in a regional hospital and, shortly after recovery, was referred to our hospital for further management.

**Figure 2:**
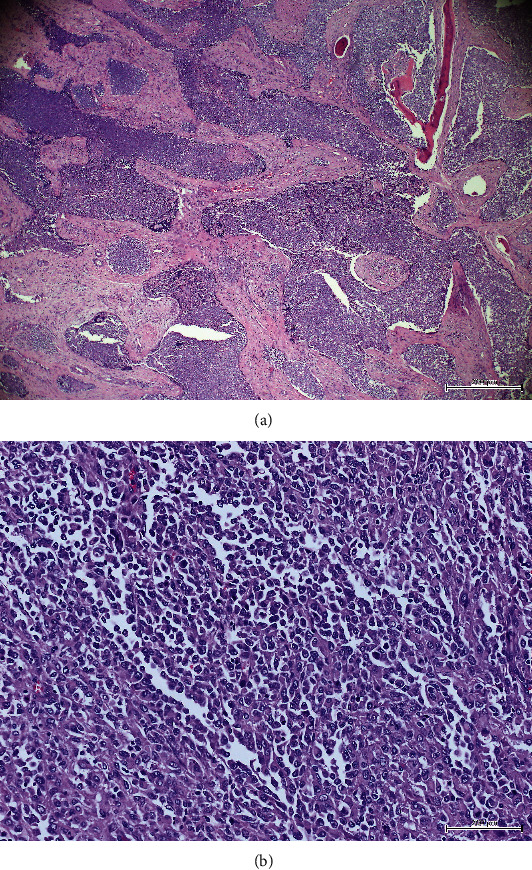
(a) Low magnification shows anastomosing sheets of infiltrating neoplasm arranged in lobules with central necrosis (hematoxylin-eosin stain, original magnification ×40). (b) High magnification shows rhabdoid morphology and brisk mitosis (hematoxylin-eosin stain, original magnification ×200).

A review of the tissue biopsies showed an infiltrating solid neoplasm with a focal alveolar pattern. Glandular, cribriform, and glomeruloid-like patterns were also noted as occasional small foci. The malignant cells were mostly rhabdoid, yet focally basaloid, clear cells, or spindled. Central necrosis and brisk mitotic activity were also evident (Figure 2). No keratinization was found. The tumor was involved in the left maxillary sinus and left upper gingivo-buccal sulcus and included foci of perineural and lymphovascular invasion. In addition, 44 out of 47 lymph nodes harbored tumor metastasis with extracapsular extension in the left cervical lymph nodes. Thus, the resection margins were involved.

**Figure 3:**
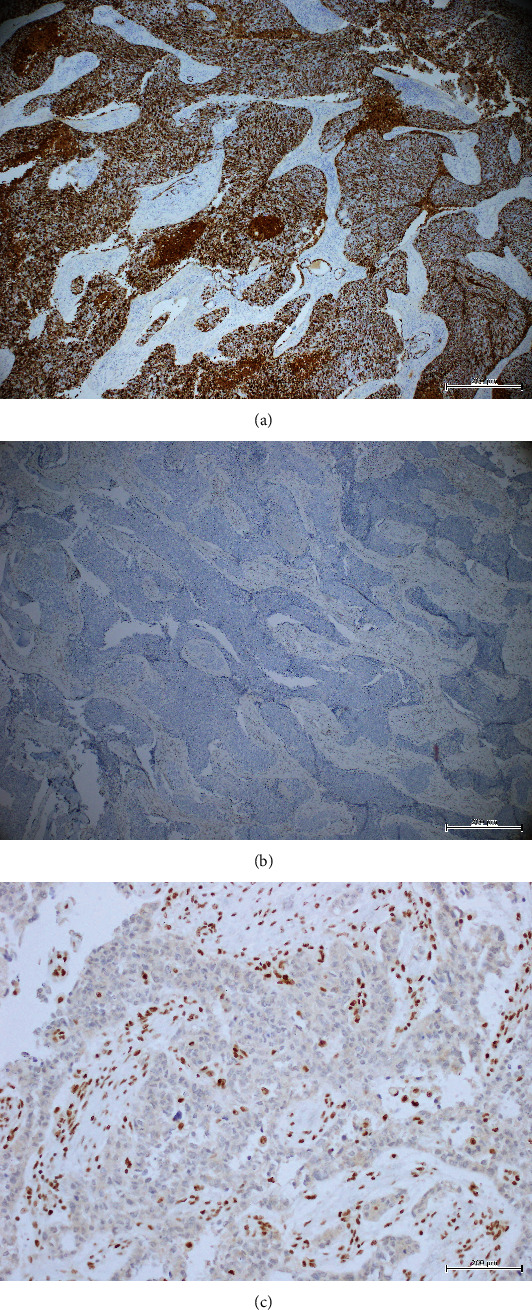
(a) The neoplastic cells show diffuse immunoreactivity for CK AE1/AE2 (original magnification (×40)). (b) The tumor exhibits complete loss of INI-1 immunostaining (×40). (c) The tumor exhibits complete loss of INI-1 immunostaining with positive control (×200).

Immunohistochemistry studies revealed that the tumor was positive for epithelial membrane antigen (EMA), pan-cytokeratins (AE1/AE3), and p40. Accordingly, WT-1, CD117, p63, CK5/6, CK7, p16, GFAP, EBV, S100, Desmin, HMB-45, Chromogranin, and Synaptophysin were all negative. INI-1 stain showed loss of nuclear expression (Figure 3). Based on the immunomorphologic characters of this tumor, it was diagnosed as SMARCB1-deficient sinonasal carcinoma.

Furthermore, the postoperative (PET-CT) scan revealed residual disease, with multiple bilateral pulmonary nodules suggestive of metastasis, and the patient was started on adjuvant radiochemotherapy. Unfortunately, the patient developed cardiac arrest and died six months after the surgery.

## 3. Discussion

The nasal tract hosts overlapping groups of histogenetically and clinically distinctive benign and malignant neoplasms in spite of its limited anatomical area.

Malignant sinonasal tract neoplasms account for less than 1% of all neoplasms of the upper aerodigestive tract [4, 5, 7] and 3% to 5% of all head and neck cancers [6, 8]. It includes carcinomas, lymphomas, and sarcomas of various types [9]. Squamous cell carcinoma is the most common pathology seen [6, 8], followed by adenocarcinoma, melanoma, and olfactory neuroblastoma [1]. In addition, a variety of aggressive tumors show undifferentiated or poorly differentiated morphology can also be seen.

The current differential diagnoses of undifferentiated or poorly differentiated sinonasal tumors include basaloid squamous cell carcinoma (SCC), sinonasal undifferentiated carcinoma (SNUC), lymphoepithelial carcinoma, small cell neuroendocrine carcinoma, poorly differentiated nonkeratinizing squamous cell carcinoma (viral and nonviral), NUT midline carcinoma [1, 10], HPV-related adenoid cystic-like carcinoma, and adamantinoma-like Ewing sarcoma [11]. Furthermore, SMARCB1-deficient sinonasal carcinoma is a rare newly described entity that falls under the diagnostic subgrouping of poorly differentiated carcinoma of the sinonasal tract [2].

SMARCB1 (INI-1) is a tumor suppressor gene located on chromosome 22q11.2 that encodes a subunit of the SWI/SNF nucleosome remodeling complex [4, 5, 12]. It is expressed universally in all nuclei of normal tissue and plays a major role in preventing tumor formation [1, 10, 13, 14]. The SMARCB1-deficient tumor family is established by inactivating mutations of the tumor suppressor gene SMARCB1 (INI-1) [8, 11, 15], which plays a role in the pathogenesis of a wide variety of tumors that share basaloid or rhabdoid morphology. Loss of INI-1 expression has emerged as an important diagnostic feature in several human malignancies including SMARCB1-deficient sinonasal carcinoma, atypical teratoid/rhabdoid tumors of the central nervous system, malignant rhabdoid tumors of the kidney and soft tissue, epithelioid sarcoma, renal medullary carcinoma, myoepithelial carcinoma of soft tissue, epithelioid malignant peripheral nerve sheath tumor, and extra skeletal myxoid chondrosarcoma [3, 8, 9].

We report a rare case of SMARCB1-deficient tumor with the typical clinicopathological characteristics reported in the previous studies including middle age at presentation and advanced stage of the disease. The morphology of the SMARCB1-deficient sinonasal carcinoma has been described in detail in the medical literature, emphasizing the highly infiltrative nature of the disease with frequent bone invasion. Grossly, the tumors have infiltrative margins with variable exophytic papillary surface components.

Histologically, most SMARCB1-deficient tumors exhibit basaloid or rhabdoid morphology. However, variety of other patterns has been described occasionally including oncocytoid, squamoid, spindle, or glandular morphology [1, 16, 17]. In our case, the malignant cells were mostly rhabdoid, with focal basaloid, clear, or spindled cell morphology. Recently, cases with predominate or focal yolk sac-like growth pattern have been also described [16, 17] expanding the morphological variation of these tumors.

Tumor necrosis and high mitotic index are a frequent finding in SMARCB1-deficient tumors as was noted in our case. However, these tumors usually lack significant pleomorphism at the cellular level. No keratinization nor dysplasia in the overlying epithelium is described in documented cases [7, 13, 16].

SMARCB1-deficient sinonasal carcinoma demonstrates strong and diffuse cytokeratin expression along with complete loss of INI-1 by immunohistochemistry. Some cases of SMARCB1-deficient sinonasal carcinoma show diffuse p63 or p40 and variable expression of p16, the former may lead to confusion with squamous cell carcinoma, and the latter will suggest HPV-related tumor. The loss of INI-1 nuclear staining excludes NUT carcinoma along with SNUC and poorly differentiated or basaloid squamous cell carcinoma [10, 15].

SMARCB1-deficient sinonasal carcinoma mostly lacks the expression of neuroendocrine markers, which excludes small-cell carcinoma and olfactory neuroblastoma. In cases with a yolk sac growth pattern, positivity for SALL-4 and glypican-3 has been reported. However, yolk sac tumors are rare in sinonasal tumor and will retain INI-1 nuclear staining [16, 17].

Patients with SMARCB1-deficient sinonasal carcinoma receive a combination of chemotherapy, radiotherapy, and surgical excision similar to other undifferentiated or poorly differentiated tumors of the sinonasal tract. However, the outcome for these patients is poor with a 5-year survival rate of 34.9% [4, 5]. Series in the literature reported distant metastasis to the lungs [18] and brain [9]. In our case, the patient developed bilateral lung metastasis.

Currently, phase II clinical trials are being conducted to investigate a specific EZH2 inhibitor therapy that could be employed as a target therapy for SMARCB1-deficient cancers in the future [7]. In the era of “precision medicine” and targeted therapies, further tumor classification based on their genetic alterations and biologic characteristics may provide entity-specific therapeutic intervention in the future and would possibly enhance the quality of life and improve survival.

## 4. Conclusion

The undifferentiated or poorly differentiated morphology is a diagnostic challenge, especially with the expansive number of uncommon entities that emerge in the sinonasal tract. The possibility of SMARCB1-deficient sinonasal carcinoma should always be included, and an adequate immunohistochemistry panel should be performed including the INI-1 stain. The accurate diagnosis of these cases is crucial for future targeted therapies.
